# Acoustic Waves Coupling with Polydimethylsiloxane in Reconfigurable Acoustofluidic Platform

**DOI:** 10.1002/advs.202407293

**Published:** 2024-10-30

**Authors:** Jeongeun Park, Beomseok Cha, Furkan Ginaz Almus, Mehmet Akif Sahin, Hyochan Kang, Yeseul Kang, Ghulam Destgeer, Jinsoo Park

**Affiliations:** ^1^ Department of Mechanical Engineering Chonnam National University Yongbong‐ro 77, Buk‐gu Gwangju 61186 Republic of Korea; ^2^ Control and Manipulation of Microscale Living Objects Center for Translational Cancer Research (TranslaTUM) Munich Institute of Biomedical Engineering (MIBE) Department of Electrical Engineering School of Computation, Information and Technology (CIT) Technical University of Munich Einsteinstraße 25 81675 Munich Germany

**Keywords:** acoustic radiation, acoustic streaming, acoustofluidics, acousto–thermal heating, wave attenuation

## Abstract

Acoustofluidics is a promising technology that leverages acoustic waves for precise manipulation of micro/nano‐scale flows and suspended objects within microchannels. Despite many advantages, the practical applicability of conventional acoustofluidic platforms is limited by irreversible bonding between the piezoelectric actuator and the microfluidic chip. Recently, reconfigurable acoustofluidic platforms are enabled by reversible bonding between the reusable actuator and the replaceable polydimethylsiloxane (PDMS) microfluidic chip by incorporating a PDMS membrane for sealing the microchannel and coupling the acoustic waves with the fluid inside. However, a quantitative guideline for selecting a suitable PDMS membrane for various acoustofluidic applications is still missing. Here, a design rule for reconfigurable acoustofluidic platforms is explored based on a thorough investigation of the PDMS thickness effect on acoustofluidic phenomena: acousto–thermal heating (ATH), acoustic radiation force (ARF), and acoustic streaming flow (ASF). These findings suggest that the relative thickness of the PDMS membrane (*t*) for acoustic wavelength (*λ*_PDMS_) determines the wave attenuation in the PDMS and the acoustofluidic phenomena. For *t*/*λ*_PDMS_ ≈ O(1), the transmission of acoustic waves through the membrane leads to the ARF and ASF phenomena, whereas, for *t*/*λ*_PDMS_ ≈ O(10), the acoustic waves are entirely absorbed within the membrane, resulting in the ATH phenomenon.

## Graphical abstract

The polydimethylsiloxane (PDMS) thickness relative to the wavelength plays a key role in wave attenuation in PDMS and can guide the design of efficient reconfigurable acoustofluidic platforms. Experimental and numerical investigations of acousto–thermal heating, acoustic radiation, and acoustic streaming suggest that the PDMS membrane thickness of approximately eight times the wavelength leads to nearly complete wave absorption within the PDMS.

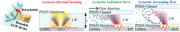

## 1 Introduction

Acoustofluidics offers a label‐free, contactless, non‐invasive, and on‐demand actuation of microfluidic lab‐on‐a‐chip platforms for numerous biomedical applications.^[^ ^1^ ^]^ In an acoustofluidic platform, surface acoustic waves (SAWs) are coupled with a microfluidic channel to actuate the fluid sample and suspended micro/nano‐objects therein. Coupling of SAWs with the microfluidic channel can i) result in acousto–thermal heating (ATH) to warm up the channel and the sample inside, ii) exert an acoustic radiation force (ARF) on the suspended objects, and/or iii) produce an acoustic streaming flow (ASF) to mix laminar co‐flowing streams.^[^ ^2^ ^]^ These SAW‐based acoustofluidic phenomena have been utilized for various applications, including polymerase chain reaction,^[^ ^3^ ^]^ thermochromic displays,^[^ ^4^ ^]^ bioparticle separation,^[^ ^5^ ^]^ cell cultures,^[^ ^6^ ^]^ patterning,^[^ ^7^ ^]^ drug delivery,^[^ ^8^ ^]^ cell lysis,^[^ ^9^ ^]^ and organism phenotyping.^[^ ^10^ ^]^

An acoustofluidic platform is composed of an interdigital transducer (IDT) patterned on a piezoelectric substrate to produce SAWs and a polydimethylsiloxane (PDMS) microchannel attached to the substrate to contain the fluidic samples. The PDMS microchannel is irreversibly bonded to the substrate by using oxygen plasma treatment of the bonding surfaces to enclose the microchannel and prevent sample leakage.^[^ ^13^, ^14^ ^]^ However, such permanent microchannel‐substrate bonding restricts the flexibility, applicability, and biocompatibility of the acoustofluidic platform. For example, the inability to replace the PDMS microchannel can lead to cross‐contamination of samples between experiments, which is detrimental to using acoustofluidic platforms in biochemical applications. Moreover, the piezoelectric substrate with a patterned IDT could only be used once, significantly increasing the experimental cost.^[^ ^11^ ^]^ Therefore, it was essential to devise a method for reversible PDMS‐substrate bonding for a detachable microchannel, where the valuable piezoelectric substrate could be reused in further experiments.

Recently, reconfigurable acoustofluidic platforms have enabled the reuse and repurposing of piezoelectric substrates by incorporating detachable and disposable microfluidic channels.^[^ ^11^, ^12^ ^]^ The microfluidic channels are sealed by a thin PDMS membrane, glass coverslip, or silicon superstrate before they are non‐permanently bonded with the piezoelectric substrates.^[^ ^13^ ^]^ The flexible nature of the PDMS membrane ensures a smooth yet reversible contact between the substrate and the microchannel. However, glass microchannels or PDMS microchannels sealed with a glass coverslip or a silicon superstrate require a coupling liquid or another thin PDMS layer at the bottom to interface with the piezoelectric substrate. In such multi‐layered acoustofluidic platforms, the SAWs originating from an IDT are radiated into the microchannel through the coupling liquid, PDMS membrane, and/or sealing superstrate. A portion of the acoustic energy is absorbed within the coupling layer, whereas the remainder of the acoustic energy is transmitted to the fluid inside the microchannel.

A PDMS membrane‐based acoustofluidic platform offers a relatively more stable acoustic energy transmission into the channel than a coupling liquid that can evaporate over time and destabilize the acoustic wave transmission. However, a viscoelastic PDMS membrane can significantly dampen the acoustic waves before they can reach the fluid inside the microchannel if the thickness of the membrane is not adequately controlled. Attenuating acoustic waves within the PDMS membranes has resulted in a noticeable increase in temperature due to the ATH phenomenon, which was harnessed to perform PCR assay,^[^ ^14^ ^]^ droplet manipulation,^[^ ^2d^ ^]^ realize digital displays,^[^ ^4^ ^]^ etc. However, unabated heating of the PDMS membrane can harm biological samples harbored within the microchannel,^[^ ^15^ ^]^ distort the microchannel,^[^ ^4^ ^]^ and reduce acoustic energy transmission to the sample. A relatively thinner PDMS membrane can reduce the acoustic wave attenuation and the ATH phenomenon while ensuring enough energy is transferred to the microchannel for the ARF and ASF phenomena to occur.^[^ ^16^ ^]^ Quantitative analysis of acoustic wave attenuation and absorption in PDMS has been limited to a low operational acoustic frequency range of 3–8 MHz due to technical challenges associated with high‐frequency waves.^[^ ^17^ ^]^ Despite the significance of the PDMS membrane in a reconfigurable acoustofluidic platform, the current literature is void of systematic design guidelines for the membrane parameters selection and a comprehensive investigation of the various acoustofluidic phenomena originating due to the variation in the membrane thickness.

In this work, we quantitatively investigated the effects of PDMS membrane thickness on ATH, ARF, and ASF phenomena in reconfigurable acoustofluidic platforms operating with the SAW frequency (*f*) range of 45–170 MHz, which is a frequently used frequency range in SAW‐based acoustofluidics.^[^ ^2c^ ^]^ For the experimental investigations, we applied infrared (IR) thermal imaging of the PDMS membrane with variable thicknesses to evaluate the ATH. We trapped microparticles inside the PDMS microchannel to indirectly quantify the ARF. We incorporated microscopic particle image velocimetry (µPIV) to analyze the ASF formed as symmetrical vortices inside the microchannel. Moreover, we developed three different numerical models to simulate the heating in the PDMS membrane, acoustic pressure field, and acoustic streaming flow field formed in the microchannel fluid domain. Based on the comprehensive evaluation within the applied SAW frequency range of 45–170 MHz, we propose that the PDMS membrane thickness (*t*) should be smaller than eight times the acoustic wavelength in PDMS (*λ*_PDMS_) to effectively utilize the ARF and ASF effects. For *t*/*λ*_PDMS_ > 8, the ATH will dominate in the reconfigurable acoustofluidic platforms with negligible ARF and ASF effects due to almost complete wave attenuation.

## 2 Results and Discussion

### 2.1 Reconfigurable Acoustofluidic Platform

**Figure 1:**
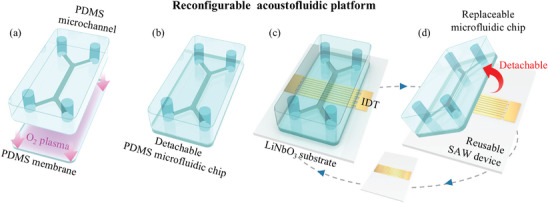
Reconfigurable acoustofluidic platform with disposable microfluidic chip and reusable SAW transducer: a) Oxygen plasma surface treatment of the lower surface of the microchannel and the PDMS membrane surface. b) Bonding of the plasma‐treated surfaces of the channel and the membrane to form a disposable microfluidic chip. c) Coupling with self‐adhesion: the disposable PDMS chip attached on top of the SAW device, viz. composed of IDT and piezoelectric substrate. d) Replacement of the used microfluidic chip and re‐use of the SAW device.

A reconfigurable acoustofluidic platform comprises a detachable PDMS microfluidic chip attached reversibly to a reusable SAW device (**Figure** **1**). The PDMS membranes with varying thicknesses were fabricated by spin‐coating a PDMS mixture on a silane‐treated Si wafer, followed by thermal curing. Since the surface roughness of the polished Si wafer is usually at the sub‐nanometer scale, the produced PDMS membranes were regarded to be at a similar scale (<nm).^[^ ^18^ ^]^ The smooth PDMS membrane permanently sealed the PDMS microchannel by oxygen plasma treatment to form a detachable microfluidic chip. The SAW device comprises an IDT deposited on a lithium niobate (LiNbO_3_; LN) piezoelectric substrate. The PDMS microfluidic chip is placed on top of the SAW device to make a reversible contact with the SAW substrate.^[^ ^11^ ^]^ The conformal contact between the PDMS microfluidic chip and the piezoelectric substrate is made by natural adhesion through van der Waals force without the need for additional coupling media or surface treatment.^[^ ^19^ ^]^ For its self‐adhesive properties, PDMS is one of the most widely used materials for flexible sensors. The PDMS membrane‐embedded flexible sensors can be attached to various substrates, including human skin, without any additional coupling media required.^[^ ^20^ ^]^ Similarly, the PDMS films adhere reversibly to the LN substrates for acoustofluidic coupling.^[^ ^2^, ^4^, ^11^, ^12^, ^21^ ^]^ Hence, the SAW device, which requires costly and labor‐intensive fabrication processes, can be reused for multiple experiments. Moreover, the PDMS microfluidic chip with a variable thickness membrane allows tunable and on‐demand transduction of acoustic energy to realize the ATH, ARF, or ASF phenomena.

### 2.2 Acoustic Waves Coupling with PDMS in Acoustofluidics

**Figure 2:**
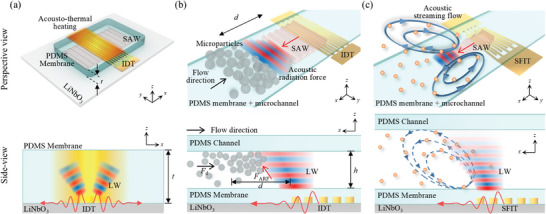
Schematic diagrams showing a) acousto–thermal heating, b) acoustic radiation force, and c) acoustic streaming flow phenomena. Perspective view (top) and *xz*‐plane side‐view (bottom).

The SAWs propagating along the substrate surface refract into the PDMS membrane following Snell's law, where the direction of the refracted wave will depend on the speed of sound in the LN substrate and the PDMS membrane.^[^ ^22^ ^]^ As the velocity of leaky longitudinal waves (LWs) in PDMS is much greater than that of the shear waves, the shear wave propagation within PDMS can be neglected for simplicity. The SAWs propagating in the ±*x*‐directions refracted into the PDMS membrane at the Rayleigh angle *θ*_R_ ≈ 16° mostly in the form of LWs. The LWs attenuate when propagating through the PDMS membrane by viscoelastic damping in PDMS, resulting in the conversion of acoustic energy into thermal energy,^[^ ^4^ ^]^ as shown in **Figure** **2**a. The acoustic attenuation length at the LN substrate‐PDMS interface (*x*_s_) is described by *x*_s_ = *ρ*_LN_ *c*_LN_ *λ*_LN_/*ρ*_PDMS_ *c*_PDMS_, where *ρ*_LN_ and *ρ*_PDMS_ are the densities of the LN substrate and PDMS, respectively; *c*_LN_ and *c*_PDMS_ are the speeds of sound in the LN substrate and PDMS, respectively; and *λ*_LN_ (=*c*_LN_/*f*) is the acoustic wavelength on the LN substrate.^[^ ^23^ ^]^ This attenuation length is determined by the material properties of both the LN and PDMS that form the interface, at which the Rayleigh surface acoustic waves propagating on the LN substrate refract into the PDMS membrane. The *x*_s _≈ 18.2*λ*_LN_^[^ ^13a^ ^]^ (µm‐scale) is smaller than the IDT aperture size (mm‐scale), and the strong acoustic attenuation at the substrate‐PDMS interface focuses the ATH effect right on top of the IDT. Moreover, the acoustic impedance mismatch at the interface determines the wave transmission and reflection at an interface. The Fresnel transmission (*T*_PDMS‐water_) and reflection (*R*) coefficients at the PDMS–water interface when a third fluid medium exists on top of the PDMS membrane, can be calculated as *T*_PDMS‐water_ ≈ 0.97 and *R* ≈ 0.03, respectively.^[^ ^24^ ^]^ As the wave transmission dominates over the wave reflection at the PDMS–water interface, we did not consider the effect of the third fluid medium. Therefore, in the present work, we used a simplified length scale of *t* for wave propagation in PDMS. The ATH phenomenon was investigated by measuring the average temperature (*T*) of the top surface of the PDMS membrane placed on the IDT. We used an IR camera to measure *T* for variable *t* and *λ*_PDMS_. As the LWs velocity (≈1000 m s^−1^) in PDMS, as specified in Table S1 (Supporting Information), is approximately ten times faster than that of the shear waves (≈100 m s^−1^),^[^ ^17a^ ^]^ we only considered the wavelength of the LWs in PDMS at varying acoustic frequency.

For a quantitative evaluation of the ARF, we used a vertical‐type acoustofluidic platform to expose suspended polystyrene (PS) microparticles to traveling SAWs through the PDMS membrane,^[^ ^21^, ^25^ ^]^ as shown in Figure 2b. The ARF acted against a flow‐induced drag force on the PS microparticles flowing inside the microchannel. The traveling SAWs on the piezoelectric substrate refracted into the fluid domain through the PDMS membrane as LWs. Despite the attenuation of LWs within the membrane, an acoustic pressure field, whose magnitude depends heavily on *t* and *λ*_PDMS_, was formed inside the microchannel. In the presence of an acoustic pressure field, the PS microparticles experienced a SAW‐induced ARF (*F*_ARF_) due to an inhomogeneous wave scattering off the particle surface. The flow‐induced drag force (*F*_d_) was counterbalanced by the *F*_ARF_ to trap the moving PS microparticles.^[^ ^21e^ ^]^ A particle trapping distance (*d*) was experimentally measured for a constant flow rate and *F*_d_, while the *F*_ARF_ magnitude gradually weakened away from the IDT and with increasing *t*. The *d* was measured from the geometric center of the trapped particle clusters to the IDT (Figure 2b). The microparticle diameter (*d*_p_) was carefully chosen to ensure a Helmholtz number (*κ* = *πd*_p_/*λ*_f_) greater than 1, where *λ*_f_ is the acoustic wavelength in the fluid. For *κ* > 1, the microparticles were dominantly influenced by ARF instead of ASF due to the Mie wave scattering.^[^ ^26^ ^]^ For efficient manipulation of the microparticles by *F*_ARF_, the *d*_p_ and *f* values were chosen to have a *κ*‐value of ≈1.5.^[^ ^27^ ^]^ The microchannel height (*h*) was intentionally designed to be significantly smaller than the acoustic attenuation length within the fluid to minimize the acoustic streaming effect.^[^ ^9^ ^]^

For a quantitative analysis of ASF, we used relatively smaller microparticles to trace the flow produced by the acoustic field gradient away from the IDT and viscous damping of the LWs within the fluid, as shown in Figure 2c.^[^ ^28^ ^]^ The acoustic wave amplitude attenuated exponentially on the surface of the LN substrate and within the viscous fluid, which led to a pressure gradient forming two symmetrical ASF vortices.^[^ ^21^, ^29^ ^]^ We intentionally used a slanted finger interdigital transducer to produce narrow‐aperture acoustic waves to produce peripheral streaming^[^ ^30^ ^]^ while suppressing the lobe streaming to facilitate the planar ASF visualization using 2D µPIV. The PS microparticles with *κ* less than 1 were used as flow tracers for µPIV analysis. For *κ* < 1, these microparticles experienced homogeneous Rayleigh scattering without a significant ARF effect and consequently followed the streaming flow field, as shown in Figure 2c. We measured the peripheral ASF‐based vorticity magnitude from the µPIV data to quantitatively evaluate the ASF for variable *t*/*λ*_PDMS_ values.

### 2.3 PDMS Thickness Effect on Acousto–Thermal Heating

**Figure 3:**
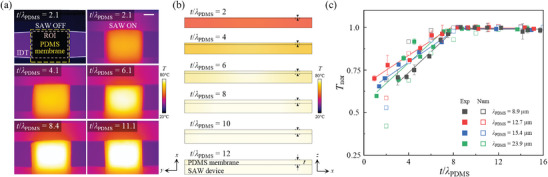
a) Experimental images of the surface temperature of PDMS membrane by acousto–thermal heating with varying PDMS membrane thicknesses at *f* = 85 MHz (*xy*‐plane). The scale bar is 500 µm. b) Numerical simulation results of the 2D temperature field (*xz*‐plane). c) Normalized PDMS surface temperature as a function of relative PDMS membrane thickness (*t*/*λ*_PDMS_) with varying SAW frequencies. Filled and empty symbols represent the experimental and numerical results, respectively. The filled square symbols indicate the average temperature with the vertical error bars indicating the standard deviation from the experiments. The solid lines represent the linear‐plateau regression fitting lines for the experimental results.

The IR thermal images demonstrate an increasing temperature trend as the PDMS membrane thickness to acoustic wavelength in PDMS ratio (*t*/*λ*_PDMS_) increased from 2.1 to 11.1 for *f* = 85 MHz at 3.15 V_rms_ (**Figure** **3**a). The temperature was measured as the average surface temperature for the region of interest (ROI), indicated by the short‐dashed rectangle in Figure 3a. Initially, the PDMS membrane was at room temperature *T*_RT_ = 20 °C without the SAWs. As the SAWs were produced by the IDT and absorbed by the PDMS membrane, the *T* increased due to the acousto–thermal heating of the viscoelastic PDMS. The *T* abruptly increased to ≈67.5 °C for *t*/*λ*_PDMS_ = 2.1 for an input voltage of 3.15 V_rms_, which confirms the PDMS‐dependent ATH phenomenon. Afterward, the *T* gradually increased with an increase in *t*/*λ*_PDMS_ from 2.1 to 8.4 (*t* was increased against a fixed *λ*_PDMS_ for a given IDT), while no significant change in *T* was observed for *t*/*λ*_PDMS_ > 8.4. The average surface temperature for ROI was measured as 67.5, 70.7, 78.1, 81.0, and 81.1 °C for *t*/*λ*_PDMS_ = 2.1, 4.1, 6.1, 8.4, and 11.1, respectively. Figure 3b depicts numerical results of acousto–thermal heating with varying *t*/*λ*_PDMS_ of 2, 4, 6, 8, 10, and 12 for *f* = 85 MHz. The numerical results matched well with the experimental observations as the *T* increased from 56.9 to 82.4 °C for *t*/*λ*_PDMS _= 2 to 8, followed by a relatively small change in *T*, that is, ±1 °C, for *t*/*λ*_PDMS _> 8. The maximum average temperature in the PDMS layer was numerically calculated as 56.9, 73.3, 80.0, 82.4, 83.2, and 83.3 °C for *t*/*λ*_PDMS_ = 2, 4, 6, 8, 10, and 12, respectively.

A normalized temperature (*T*_nor_) is defined as the relative temperature increase (*T*  −  *T*_RT_) with respect to room temperature divided by the maximum temperature increase (*T*_max_  −  *T*_RT_) for a given IDT. The *T*_nor_ was measured experimentally and calculated numerically for four different IDTs with wavelengths *λ*_PDMS_ = 8.9, 12.7, 15.4, and 23.9 µm, and corresponding frequencies *f *= 45, 70, 85, and 121 MHz, respectively (Figure 3c). We have previously noted that the ATH phenomenon could be dependent on the SAW frequency.^[^ ^3^ ^]^ Therefore, for decoupling of frequency‐dependent heating effect and varying energy conversion efficiency due to the different S_11_ values of the different IDTs, we controlled the electrical power applied to each IDT at its resonant frequency such that the maximum *T* reached ≈80 ± 3 °C for different values of *t* (10–350 µm), *f* (45–121 MHz) and *t*/*λ*_PDMS_ (0.9–16) as shown in Figure S1 (Supporting Information). The *T*_nor_ values gradually increased with an increase in *t*/*λ*_PDMS_ and plateaued at approximately eight of *t*/*λ*_PDMS_ regardless of the PDMS membrane thicknesses, applied frequencies, and *λ*_PDMS_ values (Figure 3c). We applied a linear‐plateau regression model for the normalized experimental *T*_nor_ values for the ATH phenomenon to identify the critical value of *t*/*λ*_PDMS_. As shown in Figure S2 (Supporting Information), we confirmed that the linear‐plateau regression fitting lines showed good agreement with the experimental results, yielding a high coefficient of determination (*R* ^2^ > 0.9). The breakpoint in the regression model was used to identify the critical values of *t*/*λ*_PDMS_, where the minimum *t*/*λ*_PDMS_ values were minimum with zero slope. The *T*_nor_ gradually increased and reached the plateau phase at the breakpoint, which was found to be approximately eight for *t*/*λ*_PDMS_ values (Figure S2, Supporting Information). These results suggest that the acoustic waves in PDMS were fully attenuated over the length scale of approximately eight times the acoustic wavelength as the acoustic energy was converted to heat energy in terms of ATH. An experimental 2D averaged temperature over the PDMS membrane surface (*xy*‐plane) matched well with the 1D averaged temperature obtained from the 2D numerical model (i.e., from the top boundary of the domain in *xz*‐plane). This agreement between the experimental and numerical results validates the proposed numerical model for fully coupled acoustic wave attenuation with sufficient accuracy. For example, the *T*_nor_ value gradually increased and plateaued at *t*/*λ*_PDMS_ ≅ 8 regardless of varying PDMS membrane thicknesses and four different *λ*_PDMS_ values (Figure 3a–c). A slight decrease in the experimental *T*_nor_ for *t*/*λ*_PDMS_ > 8 was due to the convective cooling at the top surface exposed to ambient air at room temperature. These results suggest that the acoustic waves in PDMS were fully attenuated over the length scale of approximately eight times the acoustic wavelength as the acoustic energy was converted to heat energy in terms of ATH.

### 2.4 PDMS Thickness Effect on Acoustic Radiation Force

**Figure 4:**
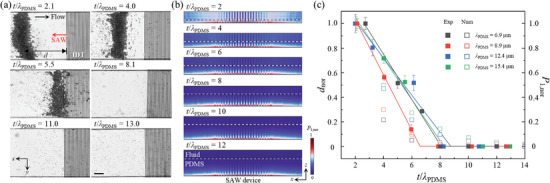
a) Experimental images of polystyrene particle trapping by acoustic radiation force with varying PDMS membrane thicknesses at *f *= 70 MHz. The scale bar is 100 µm. b) Numerical simulation results of the time‐averaged first‐order acoustic pressure field. c) Normalized particle trapping distance as a function of PDMS membrane thickness divided by acoustic wavelength in PDMS with varying frequencies. Filled and empty symbols represent the experimental and numerical results, respectively. The filled square symbols indicate the average particle trapping distance with the vertical error bars indicating the standard deviation from the experiments. The solid lines represent the linear‐plateau regression fitting lines for the experimental results.

We experimentally and numerically investigated the PDMS thickness effect on ARF as the acoustic pressure field within the microchannel attenuated away from the IDT. At a fixed flow condition, the flow‐induced drag force acting on the suspended particle remained constant regardless of the varying PDMS membrane thicknesses. On the other hand, the SAW‐induced ARF magnitude decreased with increasing PDMS membrane thickness due to the wave attenuation in PDMS. The acoustic field intensity was indirectly evaluated in the microparticle trapping experiments by measuring the particle trapping distance (*d*) from the IDT front end to the trapped particle cluster center (**Figure** **4**a).

The PDMS microchannel was aligned parallel to the direction of acoustic wave propagation (Figure 4a). The PS microparticles were exposed to SAWs with 70 MHz frequency and input voltage of 4.24 V_rms_ for variable *t*/*λ*_PDMS_ values of 2.1, 4.0, 5.5, 8.1, 11.0, and 13.0, respectively. The particle suspension solution was injected into the microchannel at a rate of 3 µl min^−1^ for all the experiments for quantitative investigation of the *t* effect on acoustic radiation. As the low‐velocity flow condition allowed the flow‐induced drag force to be small, we assumed that if the particles were not trapped by the acoustic radiation force, the acoustic field intensity was negligibly small. On the onset of the SAW generation from the IDT (from right to left in Figure 4a), the particles flowing by Stokes drag experienced the SAW‐induced ARF in the +*x* and +*z*‐directions. The horizontal component served as a virtual obstacle to the particle flow, while the vertical component caused the particles to move upward, resulting in the particle entrapment near the microchannel ceiling. The particles were trapped at the quasi‐equilibrium location where the drag and radiation forces were counterbalanced in the microchannel. The particle trapping distance was measured as *d* = 722.7, 518.7, and 379.6 µm for *t*/*λ*_PDMS_ = 2.1, 4.0, and 5.5, respectively. The *d* value serves as an indirect measure of the acoustic pressure field intensity. With increasing *t*/*λ*_PDMS_ from 2.1 to 5.5, an increasing wave attenuation resulted in a gradually diminishing acoustic pressure field formed inside the microchannel, leading to a decreasing trend for *d*. For *t*/*λ*_PDMS_ = 8.1, 11.0, and 13.0, the particles could not be trapped due to an insufficient acoustic pressure field caused by significant wave attenuation within comparatively thicker PDMS membranes.

A normalized first‐order acoustic pressure (*P*_1,nor_) for *t*/*λ*_PDMS _= 2–12 at *f* = 70 MHz was plotted in the PDMS membrane and microfluidic channel using the numerical model (Figure 4b). A strong acoustic pressure field was formed for a relatively small *t*/*λ*_PDMS_ value of 2, corresponding to particles trapped by the ARF farthest from the IDT, as indicated in the experimental case of *t*/*λ*_PDMS_ = 2.1 (Figure 4a). As the *t*/*λ*_PDMS_ increased from 2 to 6, the increased wave attenuation in the PDMS membrane resulted in the weakened acoustic pressure field intensity in the fluid domain. These numerical results agreed well with the decreasing *d* value as the *t*/*λ*_PDMS_ increased from 2.1 to 5.5 in the experiments (Figure 4a). For *t*/*λ*_PDMS_ greater than 8, the acoustic pressure intensity approached zero because of complete wave attenuation in the PDMS membrane, whose thickness was sufficiently thick compared to the acoustic wavelength in PDMS. An insignificant acoustic field intensity for *t*/*λ*_PDMS_ larger than 8 substantiated the experimental results with negligible particle trapping. These results suggest that the PDMS membrane thickness plays a critical role in wave attenuation and, consequently, in forming the acoustic pressure field in the microchannel fluid domain.

Figure 4c represents the normalized trapping distance (*d*_nor_ = *d*/*d*_max_) as a function of *t*/*λ*_PDMS_ for varying *λ*_PDMS_ of 6.9, 8.9, 12.4, and 15.4 µm (corresponding *f* = 70, 87, 121, and 154 MHz, respectively). The *d*_max_ value was defined as the maximum *d* value for each case of *λ*_PDMS_. All the experimental results show that the particles were no longer trapped with *d*_nor_ = 0 for *t*/*λ*_PDMS_ > 8 in all *λ*_PDMS_ conditions. It indicates a negligible acoustic field was formed in the microchannel fluid domain as all the wave energy was converted to heat inside the PDMS membrane, similar to the acousto–thermal heating phenomenon in Figure 4c. The utilization of a 2D numerical model, along with the absence of frequency‐dependent material properties, contributes to the convergence of numerical and experimental results.

### 2.5 PDMS Thickness Effect on Acoustic Streaming Flow

**Figure 5:**
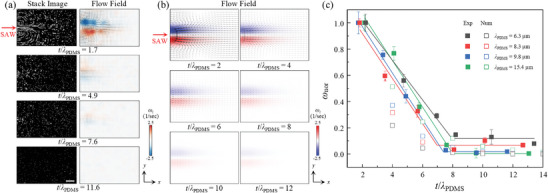
a) Experimental images of flow tracing particles and flow field by acoustic streaming with varying PDMS membrane thicknesses at *f *= 110 MHz. The scale bar is 100 µm. b) Numerical simulation results of the Eckart streaming flow field. The arrows indicate the streaming velocity vector field, and the color contour map represents the vorticity magnitude. c) Normalized SAW‐induced vorticity as a function of PDMS membrane thickness divided by acoustic wavelength in PDMS with varying frequencies. Filled and empty symbols represent the experimental and numerical results, respectively. The filled square symbols indicate the average maximum vorticity with the vertical error bars indicating the standard deviation from the experiments. The solid lines represent the linear‐plateau regression fitting lines for the experimental results.

The attenuation of acoustic waves in PDMS was further characterized by evaluating the ASF induced by the propagation of waves within the fluid domain. We conducted experimental and numerical investigations on the ASF fields for variable *t*/*λ*_PDMS_. **Figure** **5**a illustrates the stacked images of the flow tracing microparticles and 2D flow field obtained by µPIV analysis for *t*/*λ*_PDMS_ = 1.7, 4.9, 7.6, and 11.6 for *f* = 110 MHz at 0.25 V_rms_. We also calculated the vorticity (*ω_z_*) in the *xy*‐plane for a quantitative comparison of the ASF intensity. The experimental ASF velocity magnitude and *ω*_z_ decreased as the PDMS membrane thickness increased, leading to a significant wave attenuation in the PDMS membrane. As observed in the ATH and ARF experiments, a complete wave attenuation for *t*/*λ*_PDMS_ > 8 resulted in the absence of the acoustic field formed inside the microchannel and a negligible acoustic streaming flow field. The maximum vorticity in the flow field was measured as 1.980, 0.610, 0.059, and 0.062 s^−1^ for *t*/*λ*_PDMS_ = 1.7, 4.9, 7.6, and 11.6, respectively. Moreover, we performed a parametric analysis of the ASF, based on a 3D numerical model described in **Figure** 7b, by varying the *t*/*λ*_PDMS_ values from 2–12 at *f* = 110 MHz (Figure 5b). Consistent with the experimental results, a monotonic decrease in the ASF velocity magnitude and vorticity was observed with increasing *t*/*λ*_PDMS_.

The experimental and numerical results were combined for all *λ*_PDMS_ values of 6.3, 8.3, 9.8, and 15.4 µm, where the normalized vorticity (*ω*_nor_) was plotted as a function of *t*/*λ*_PDMS_ (Figure 5c). The *ω*_nor_ was calculated by dividing *ω*_z_ with the maximum vorticity (*ω*_max_) for each *t*/*λ*_PDMS_. With higher *t*/*λ*_PDMS_ values and wave attenuation, the ASF field weakened with a decrease in *ω*_nor_. For *t*/*λ*_PDMS_ > 8, both numerical and experimental results showed that the ASF vorticity was negligible. The experimental vorticity of the acoustic streaming flow field was considered negligibly small when the normalized vorticity fell below ≈0.1. In Figure 5c, the experimental vorticity values under the *t*/*λ*_PDMS_ > 8 conditions were measured to be not completely zero. The non‐zero normalized vorticity could be attributed to the thermophoresis of the tracing particles due to acousto–thermal heating. This interesting finding can serve as a useful design guideline for the PDMS membrane thickness in reconfigurable acoustofluidic platforms depending on varying working acoustic frequencies. For the acousto–thermal applications, the PDMS membrane should be sufficiently thick as *t* > 8*λ*_PDMS_ for complete wave absorption. On the contrary, for optimal performance in acoustofluidic applications based on radiation or streaming, the PDMS membrane thickness should be small compared to the acoustic wavelength, such as *t* < 8*λ*_PDMS_. Moreover, if a double‐thickness PDMS membrane is used, one thickness in the acoustic regime (*t*/*λ*_PDMS _< 8) and the other in the acousto‐thermal regime (*t*/*λ*_PDMS _> 8), in the proposed reconfigurable acoustofluidic platforms, the acoustic radiation or streaming effects could be simultaneously induced together with acousto–thermal heating in a single chip.

### 2.6 Acoustic Waves with Power‐Law Attenuation

**Figure 6:**
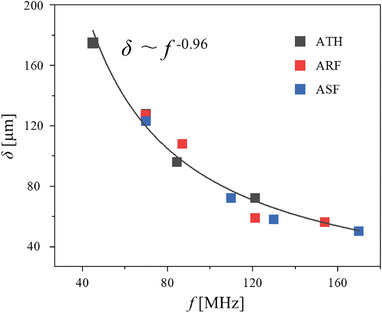
Power‐law fitting plot for frequency and penetration depth (defined here as PDMS thickness where most of the acoustic waves are absorbed) for each experiment: ATH, ARF, and ASF.

The acoustic wave attenuation length scale can be quantified as a penetration depth (*δ*), which is known to follow the power law of *δ* ∝ *f* ^−^ *^γ^*, where *γ* is a real non‐negative material parameter.^[^ ^3^, ^31^ ^]^ In each of the ATH, ARF, and ASF experiments, we considered *δ* as the thinnest *t* needed for a complete wave attenuation for any given *f* (**Figure** **6**). Therefore, the smallest *t* values to have *T*_nor_ ≅ 1 in the ATH experiments (Figure 3c), *d*_nor_ ≅ 0 in the ARF experiments (Figure 4c), and *ω*_nor_  ≅ 0 in the ASF experiments (Figure 5c) were used as *δ* in the *δ–f* plot (Figure 6). In the ATH experiments, the region where *T*_nor_ ≅ 1 was identified at which the minimum *t*/*λ*_PDMS_ values were minimum with zero slope in the linear‐plateau fitting lines (Figure S2, Supporting Information). Similarly, in the ARF and ASF experiments, the region where *d*_nor_ ≅ 0 (Figure S3, Supporting Information) and *ω*_nor_ ≅ 0 (Figure S4, Supporting Information), respectively, were found at which the *t*/*λ*_PDMS_ values reached minimum with zero slope in the linear‐plateau fitting lines. At the critical *t*/*λ*_PDMS_ conditions, the particles were not trapped with negligible radiation force in the ARF experiments while the normalized vorticity fell below ≈0.1 in the ASF experiments. The PDMS thicknesses, indicating the penetration depths (i.e., *t* =* δ*), were deduced as 175, 128, 96, and 72 µm for *f* = 45, 70, 85, and 121 MHz in the ATH experiments, 127, 108, 59, and 56 µm for *f* = 70, 87, 121, and 154 MHz in the ARF experiments, and 123, 72, 58, and 50 µm for *f *= 70, 110, 130, and 170 MHz in the ASF experiments, respectively. The *γ* parameter from the power‐law fitting of *δ–f* plot was ≈0.96 in the present study, which was slightly higher than *γ* = 0.7 estimated previously only by the ATH experiments using a low‐resolution infrared camera.^[^ ^3^ ^]^ This *γ* value found in the present study can serve as a useful guideline to calculate the appropriate PDMS membrane thickness of reconfigurable acoustofluidic platforms depending on acoustic frequency.

We examined the previously reported reconfigurable acoustofluidic platforms based on our findings of the critical *t*/*λ*_PDMS_ value (**Table** **1**). We listed the *f* and *t*/*λ*_PDMS_ values to categorize these reported platforms based on the dominant acoustofluidic phenomena, for example, ATH, ARF, and/or ASF. For the acoustofluidic platforms utilizing ARF or ASF, the *t*/*λ*_PDMS_ value was smaller than 8. On the other hand, the ATH‐based platforms had *t*/*λ*_PDMS_ ≳ 8. These observations were consistent with our experimental and numerical results described above.

**Table 1:** Previous literature on reconfigurable acoustofluidic platforms.

Physical regimes	Acoustic radiation force and acoustic streaming flow											Acousto–thermal heating		
*f* [MHz]	33	19	65–280	19	25–99	161–171	72	100–121	110	129	97	20–45	15–97	70–95
*t*/*λ*_PDMS_	0.4, 0.6	0.6	0.9–3.9	1.2–1.3	1.2–4.6	2.2–2.4	2.5	4.6–5.6	0.9	4.8	6.7	7.4–16.7	9.7–63.0	16.6–22.5
Ref.	[16a]	[13b]	[32]	[16b]	[13a]	[33]	[21e]	[11]	[16c]	[21c]	[21b]	[4]	[3]	[2d]

## 3 Conclusion

In this study, we proposed the quantitative design guidelines for the PDMS membrane thickness in reconfigurable acoustofluidic platforms depending on working acoustic frequency. We experimentally and numerically analyzed the ATH, ARF, and ASF phenomena by varying PDMS thicknesses and acoustic frequencies to indirectly investigate acoustic attenuation in a PDMS membrane. In the ATH investigation, we observed heat generation due to acoustic wave absorption in a PDMS membrane with varying thickness as acoustic waves were coupled from an IDT to the PDMS at various frequencies. As the acoustic waves attenuated, the PDMS surface temperature increased with the membrane thickness and finally stabilized. We confirmed that propagating waves in PDMS are fully attenuated over a length scale of approximately eight times the acoustic wavelength. In the investigation of ARF and ASF, the increased wave attenuation in the PDMS membrane led to a decrease in the acoustic pressure field intensity in the fluid domain. The ARF and ASF effects were only dominant for a relatively thinner PDMS membrane. After the wave was attenuated to a length scale of approximately eight times the acoustic wavelength in PDMS, the acoustic field nearly approached zero, and the ARF/ASF effects were negligible. Based on the findings, we conclude that the PDMS membrane thickness relative to the acoustic wavelength plays a crucial role in the working mechanism of wave attenuation and, thus, serves as a critical design rule for reconfigurable acoustofluidic platforms.

## 4 Experimental Section

### Surface Acoustic Wave Generation

The SAW device was fabricated by depositing a bimetallic electrode composed of Cr (300 Å) and Au (1000 Å) onto a 500‐µm‐thick, 128° Y‐cut, X‐propagating LN wafer (MTI Korea) via e‐beam evaporation and lift‐off process using negative photoresist (NR9‐3000PY, Futurrex, Inc.). For efficient generation of Rayleigh‐type SAWs along the substrate, *X‐*direction of the 128° Y‐cut, X‐propagating LN wafers were utilized since the *X*‐direction velocity is 200% higher than that in the *Y‐*direction on the 128° Y‐cut, X‐propagating LN wafers.^[^ ^34^ ^]^ The resonant frequencies of each IDT were determined using a vector network analyzer (E5071B, Agilent Technologies). Sinusoidal alternating current signals were applied using a power supply (EX50‐24, ODA Technology), signal generator (N9310A, Keysight), and amplifier (ZHL‐100W‐GAN+, Mini‐Circuits) to generate SAW. All the electrical voltage values provided in this study were measured by an oscilloscope (MSO8104A, Agilent Technologies) after the applied AC signals produced from a signal generator were amplified by an RF amplifier with a typical gain of 42 dB.

### Disposable Microfluidic Chip Fabrication

Each PDMS microchannel was fabricated by photolithography and soft lithography processes. The PDMS membrane was fabricated through the spin‐coating process of an uncured solution of PDMS base and curing agent (Sylgard 184A and 184B, Dow Corning) in a 10:1 (w/w) ratio using a spin coater (SF‐100NA, BGK), followed by thermal curing at 80 °C for 1 h. The PDMS microchannel was irreversibly bonded to a PDMS membrane through oxygen‐plasma treatment (Covance, Femto Science) to form a disposable microfluidic chip.

### Acousto–Thermal Heating Experiments

In the ATH experiments, the SAW device was positioned on a Peltier cooling system (TEC Controller, Photondays Co.) to maintain the stage temperature at 20 °C and to decouple the system from surrounding fluctuations.^[^ ^35^ ^]^ The temperature increase resulting from ATH within the PDMS membrane was quantified by capturing thermal images using an infrared camera (A325sc, Teledyne FLIR) with a macro lens (FLIR Close‐Up Lens 1× (25 µm), Teledyne FLIR) on five separate occasions. For a range of thickness, such as 27–349, 21–230, 12–192, and 27–140 µm, four different IDTs were used with resonant frequencies of 45, 70, 85, and 121 MHz, corresponding to acoustic wavelength in PDMS of 23.9, 15.4, 12.7, and 8.9 µm, respectively. *λ*_PDMS_ was calculated by dividing the speed of longitudinal waves in PDMS (1076.5 m s^−1^) with the resonant frequency of the IDT.

### Acoustic Radiation Force and Acoustic Streaming Flow Experiments

In the ARF and the ASF experiments, the fluids and microparticles were injected into the microchannel using a syringe pump (Fusion 4000, Chemyx). The ARF and ASF experimental results were recorded using a high‐speed camera (VEO 710L, Phantom Ametek) mounted on an inverted microscope (IX73, Olympus) on five separate occasions. In the ARF experiments, PS microparticles (Thermo Fisher Scientific, Inc.) were used with four different *d*_p_ (11.1, 8.9, 6.1, and 4.6 µm) for variable *t* (32–200, 25–157, 19–113, and 19–84 µm), *f* (70, 87, 121, and 154 MHz) and *λ*_PDMS_ (15.4, 12.4, 8.9, and 6.9 µm) values, respectively. The Helmholtz number in the ARF experiments was estimated to be *κ* = 1.65, 1.64, 1.57, and 1.50 for *f* = 70, 87, 121, and 154 MHz and *d*_p_ = 11.1, 8.9, 6.1, and 4.6 µm, respectively. The height and width of the rectangular microchannel in the ARF experiments were 50 and 500 µm, respectively. In the ASF experiments, the red fluorescent particles (Thermo Fisher Scientific, Inc.) were used with the microparticle diameter (*d*_p _= 1 µm) for variable *t* (33–201, 17–114, 15–105, and 14–85 µm), *f* (70, 110, 130, and 170 MHz) and *λ*_PDMS_ (15.4, 9.8, 8.3, and 6.3 µm) values, respectively. The Helmholtz number in the ASF experiments was estimated to be *κ* = 0.15, 0.23, 0.28, and 0.36 for *f* = 70, 110, 130, and 170 MHz and *d*_p_ = 1 µm, respectively. The height and width of the rectangular microchannel in the ASF experiments were 100 and 750 µm, respectively. The ASF experiments were conducted under fully stopped‐flow conditions after injecting microparticles.

### Microscopic Particle Image Velocimetry to Analyze the ASF

For µPIV analysis, experimental images were captured at 50 fps using a 532 nm laser (MGL‐F‐532, Changchun New Industries Optoelectronics Technology). In the µPIV settings stage, the PIV algorithm with FFT window deformation was used. The vorticity of the flow was calculated by the 2D cross‐correlation using an interrogation window of 64 × 64 pixels^2^ with 50% overlap and 32 × 32 pixels^2^ with 50% overlap by utilizing PIVlab in MATLAB. For µPIV analysis, the Stokes number (St = *τ*_p_/*τ*_f_) was approximately calculated as St = 0.1 × 10^−9^–1.3 × 10^−7^, 0.4 × 10^−8^–1.9 × 10^−8^, 0.2 × 10^−8^–1.6 × 10^−8^, and 0.7 × 10^−9^–1.6 × 10^−8^ for *f* = 70, 110, 130, and 170 MHz, respectively; where *τ*_p_ = (1/18)*ρ*_p_ *d*_p_^2^/*μ*_f_ is the particle response time, *τ*_f_ = *L*/*V* is the flow time scale, *ρ*_p_ is the density of the particles, *μ*_f_ is the dynamic viscosity of the fluid, *L* is the characteristic length of the microchannel, and *V* is the flow velocity obtained from the PIV results.

### Numerical Section

**Figure 7:**
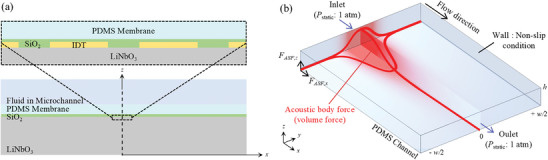
Numerical schematics to observe ATH, ARF, and ASF. a) 2D model for ATH and ARF. b) 3D model for ASF.

Acoustofluidic platforms were complex, involving various physical phenomena and scales, ranging from small‐scale SAWs to large‐scale bulk fluid dynamics. Numerous multiphysics simulation models were developed to comprehend the dynamics of such platforms and elucidate experimental results.^[^ ^36^ ^]^ In this study, three numerical models (**Figure** **7**) were developed to observe ATH, ARF, and ASF phenomena for different acoustofluidic platforms by coupling several modules in COMSOL Multiphysics software, such as Electrostatics, Solid Mechanics, Pressure Acoustics, Heat Transfer, and Laminar Flow modules.

### 2D Coupled Models for ATH

A 2D fully coupled numerical model, comprising LN substrate, metal electrodes, SiO_2_ layer, PDMS membrane, and fluid within the microchannel, was developed to conduct a study in the frequency domain (Figure 7a). The wave attenuation in the PDMS membrane, for variable acoustic wavelengths and membrane thicknesses, was investigated to understand its effects on ATH and acoustic pressure field by utilizing the Electrostatics, Solid Mechanics, and Piezoelectric Effect modules in COMSOL Multiphysics. The SAWs were produced in the model due to the elastic deformation of the piezoelectric substrate when an electric field was applied to the IDT. It was assumed that PDMS was thermally conductive and viscous, which was validated to accurately predict the physical phenomena in the SAW‐PDMS acoustofluidic devices.^[^ ^37^ ^]^ The pressure acoustics simulations were performed to determine the first‐order pressure field (*P*_1_) within the PDMS membrane by applying the Acoustic–Structure Boundary Multiphysics module to couple the LiNbO_3_ substrate, PDMS membrane, and microchannel domains. The Heat Transfer in the Solids and Fluids module was also used to calculate the stationary temperature field of the entire platform, considering heat generation due to the wave attenuation within the PDMS membrane. The boundary conditions of the model were established based on specific assumptions: the upper and side boundaries were assumed to be free and surrounded by air, and the lower boundary was set as fixed and perfectly insulated for the Solid Mechanics and Heat Transfer modules. In the numerical simulation of ATH, heat generation was measured by varying the PDMS membrane thicknesses on a SAW device composed of an IDT, substrate, and silicon‐dioxide layer. The frequencies used in the simulations were 45, 70, 85, and 121 MHz, with corresponding PDMS membrane thicknesses ranging from 48–383, 31–246, 25–203, and 18–142 µm, respectively. Eleven electrode pairs were used to simulate IDTs. The convective heat transfer coefficient of free air and the conductive heat transfer coefficient of the PDMS medium used in the module were taken as 25 W m^−^^2^ K^−1[^ ^38^ ^]^ and 0.27 W m^−1^ K^−1^,^[^ ^39^ ^]^ respectively.

### 2D Coupled Models for ARF

For the ARF investigation, the microchannel fluid domain was additionally introduced into the model described above for the ATH simulation (Figure 7a), while excluding the Heat Transfer module and including the Pressure Acoustics module instead. A frequency domain study was conducted to calculate the first‐order acoustic pressure field for the PDMS and fluid domains at varying frequencies and PDMS membrane thicknesses. These numerical models enabled comparison with the experimental results showing particle trapping within the microchannel because the ARF field was found to predominantly depend on the pressure gradients in the first‐order acoustic field.^[^ ^40^ ^]^ The boundary conditions of the fluid domain were set as impedance boundary conditions, with PDMS at the upper boundary and water at the side boundaries. An input voltage of 10 V was applied for all simulations, and the pressure field was normalized for comparative evaluation under varying conditions. The time‐averaged first‐order acoustic pressure field, normalized by the maximum pressure, was calculated from the 2D thermo‐viscous numerical model for varying *t*/*λ*_PDMS_ and acoustic frequencies. The frequencies used in the simulations were 70, 87, 121, and 154 MHz, and their corresponding PDMS membrane thicknesses ranged from 31–215, 25–173, 18–125, and 14–98 µm, respectively.

### 3D ASF Model

A 3D model (Figure 7b) was developed to observe the Eckart streaming flow fields under various conditions for the ASF investigation. The LWs propagating within a fluid medium experienced attenuation due to viscous damping, resulting in the generation of the ASF field.^[^ ^41^ ^]^ The ASF induced by the wave attenuation in fluids could be numerically modeled by incorporating a body force (${\vec{F}}_{ASF}$) term in the Navier–Stokes equation. In cases where standing wave formation was negligible due to minimal reflection of traveling LWs, a momentum flux or body force emerged in the direction of wave attenuation.^[^ ^42^ ^]^ Kiebert et al.^[^ ^43^ ^]^ derived a simplified body force representation, which could be mathematically described as:

(1) $${\vec{F}}_{ASF}=\rho_{f}\;\omega^{2}u_{0}e^{\left(-2\left(b_{z}x+b_{y}y\right)\right)}\frac{\left(\vec{b}\vec{b}\cdot \vec{b}\;-\;\vec{k}\vec{b}\cdot \vec{k}\right)}{{\left(-\vec{b}+\vec{k}\right)}^{2}}$$

where *ρ*_f_ is the fluid density, *ω* is the angular frequency, *u*_0_ is the amplitude, and $\vec{k}$ and $\vec{b}$ are real and imaginary parts of the complex wave vector, respectively. Equation (1) was integrated into the Laminar Flow module of COMSOL Multiphysics as a body force to solve the Navier–Stokes equation under laminar flow assumptions.^[^ ^26^ ^]^ Second‐order velocity and vorticity fields arising from traveling SAWs propagation for different thicknesses of the PDMS membrane and various frequencies were determined. The wave amplitude (*u*_0_) for each membrane thickness and frequency were taken from the 2D fully coupled simulations (described above). The boundary conditions were set as follows: the upper, side, and lower boundaries were defined as a wall with a no‐slip condition, and the boundaries in the flow direction were considered open to account for a quiescent flow. The simulation parameters and simulation models are provided in Table S1 (Supporting Information). In the numerical simulation of ASF, the vorticity in the *xy*‐plane was calculated for variable PDMS membrane thickness and acoustic frequency. The frequencies used in the simulations were 70, 110, 130, and 170 MHz, with corresponding PDMS membrane thicknesses ranging from 31–215, 20–137, 17–116, and 13–89 µm, respectively.

## Conflict of Interest

The authors declare no conflict of interest.

## Supporting information



Supporting Information

## Data Availability

The data that support the findings of this study are available from the corresponding author upon reasonable request.
