# Supplementary material for: Acoustic Waves Coupling with Polydimethylsiloxane in Reconfigurable Acoustofluidic Platform
Source: Adv Sci (Weinh). 2024 Oct 30;11(47):2407293. doi: 10.1002/advs.202407293 (PMC11653602; doi:10.1002/advs.202407293)
Supplement: Supplementary file 1 — Supporting Information [file ADVS-11-2407293-s001.docx]

Supporting Information

Acoustic Waves Coupling with Polydimethylsiloxane in Reconfigurable Acoustofluidic Platform

Jeongeun Park, Beomseok Cha, Furkan Ginaz Almus, Mehmet Akif Sahin, Hyochan Kang, Yeseul Kang, Ghulam Destgeer*, and Jinsoo Park*

| **Physical Regimes** | | **ATH** | **ARF** | **ASF** |
| --- | --- | --- | --- | --- |
| **Geometrical Parameters** | Channel thickness [µm] | 50 | | 100 |
|  | Substrate thickness [µm] | 300 | |  |
|  | Silicon dioxide thickness [µm] | 0.4 | |  |
|  | Electrode thickness [µm] | 0.2 | |  |
|  | Channel length [µm] | 4000 | | 2000 |
| **Material Parameters** | PDMS speed of sound [m s^-1^] | 1076.5 | | |
|  | Water speed of sound [m s^-1^] | 1500 | | |
|  | PDMS density [kg m^-3^] | 970 | | |
|  | Water density [kg m^-3^] | 1000 | | |
|  | Silicon dioxide density [kg m^-3^] | 2200 | | |
|  | Substrate density [kg m^-3^] | 4700 | | |
|  | PDMS dynamic viscosity [Pa s] | 0.0794 | | |
|  | PDMS bulk viscosity [Pa s] | 0.0194 | | |
| **Electrostatic & Thermal Parameters** | Input voltage [V] | 3.3 @*f* = 121 MHz  4.0 @*f* = 85 MHz  4.5 @*f* = 70 MHz  6.0 @*f* = 45 MHz | 10 | 5 |
|  | Thermal conductivity [W m^-1^ K^-1^] | 0.027 | | |
|  | Convective heat transfer coefficient [W m^-2^ K^-1^] | 25 | | |
|  | Number of electrode pairs | 11 | | |

**Table S1.** The simulation parameters used in the numerical analysis of acousto-thermal heating (ATH), acoustic radiation force (ARF), and acoustic streaming flow (ASF) phenomena.

**
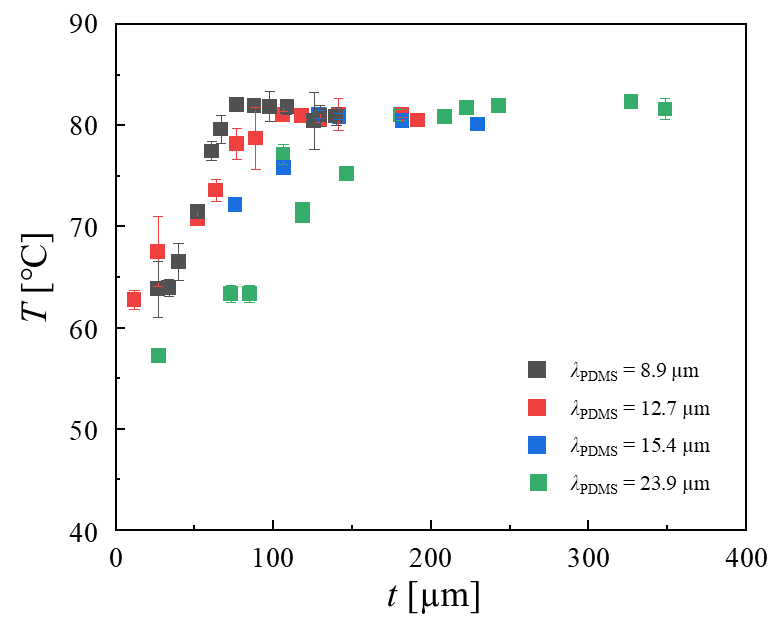
**

**Figure S1.** The ATH experimental results of PDMS surface temperature (*T*) as a function of relative PDMS membrane thickness (*t*) with varying SAW frequencies (*f*).

**
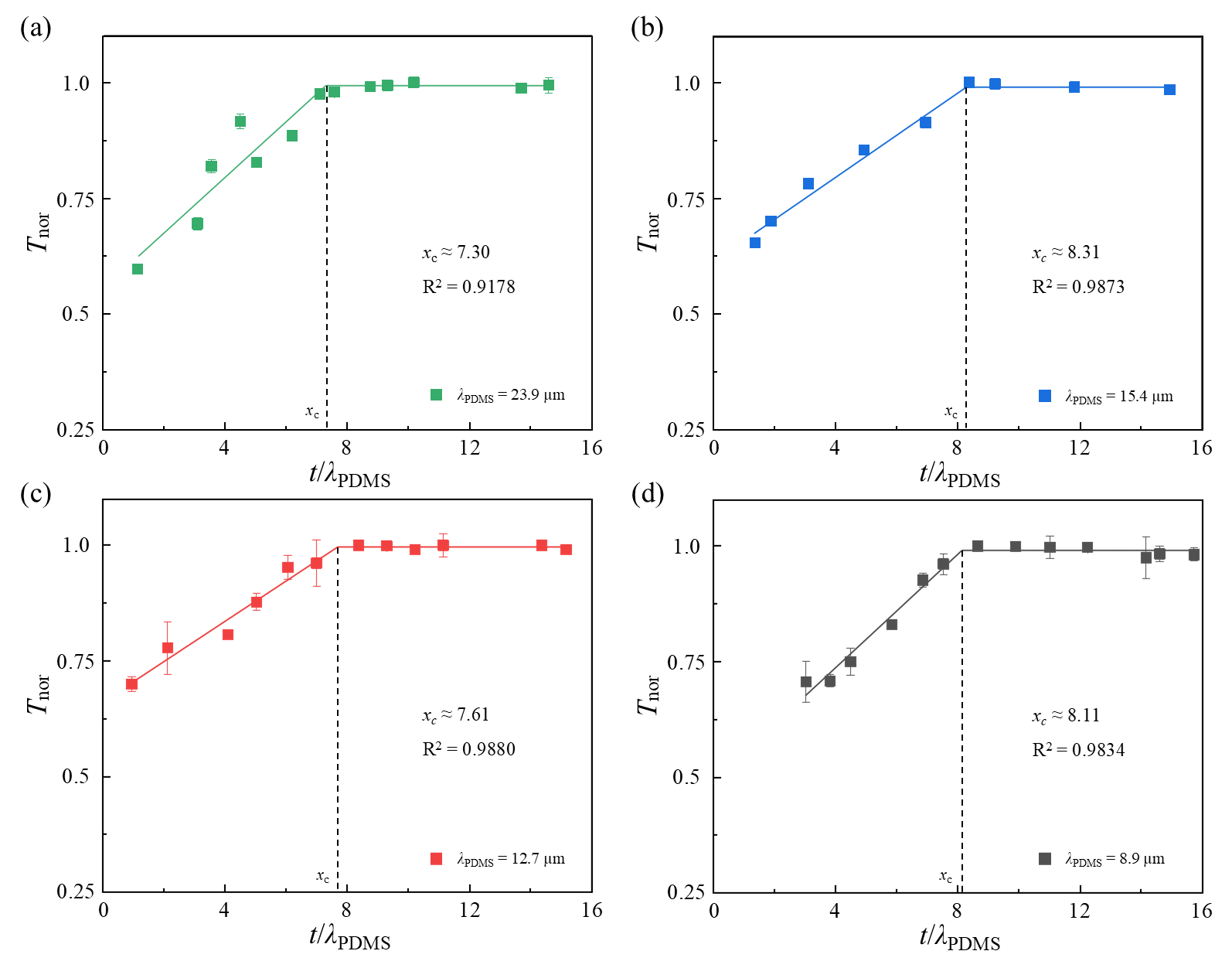
**

**Figure S2.** Linear-plateau regression results of the ATH experiment for normalized *T* as a function of *t* to acoustic wavelength in PDMS ratio (*t*/*λ*_PDMS_) at (a) *λ*_PDMS_ = 23.9 µm (*f* = 45 MHz), (b) *λ*_PDMS_ = 15.4 µm (*f* = 70 MHz), (c) *λ*_PDMS_ = 12.7 µm (*f* = 85 MHz), (d) *λ*_PDMS_ = 8.9 µm (*f* = 121 MHz).

**
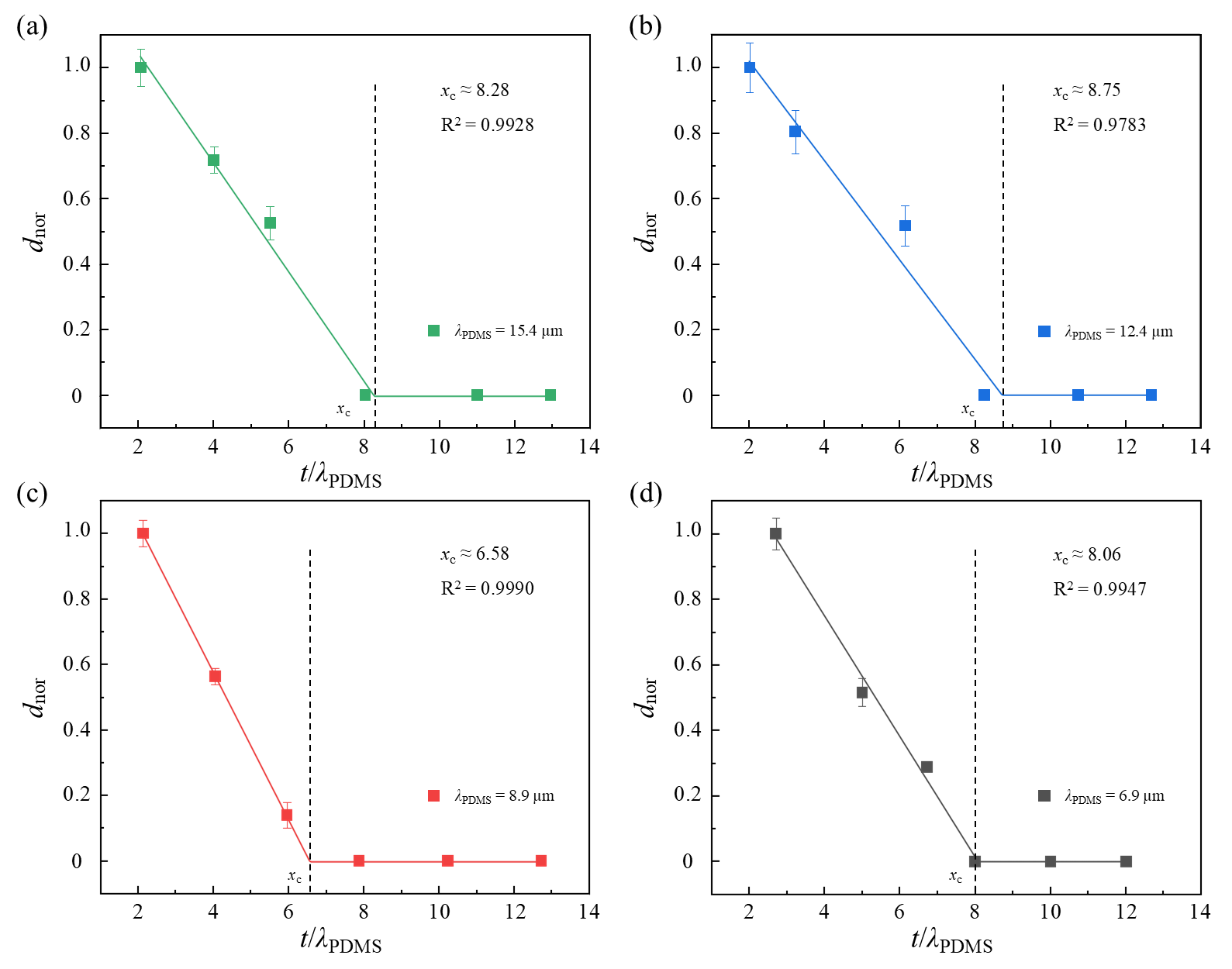
**

**Figure S3.** Linear-plateau regression results of the ARF experiment for normalized particle trapping distance (*d*_nor_) as a function of *t*/*λ*_PDMS_ at (a) *λ*_PDMS_ = 15.4 µm (*f* = 70 MHz), (b) *λ*_PDMS_ = 12.4 µm (*f* = 87 MHz), (c) *λ*_PDMS_ = 8.9 µm (*f* = 121 MHz), (d) *λ*_PDMS_ = 6.9 µm (*f* = 154 MHz).

**
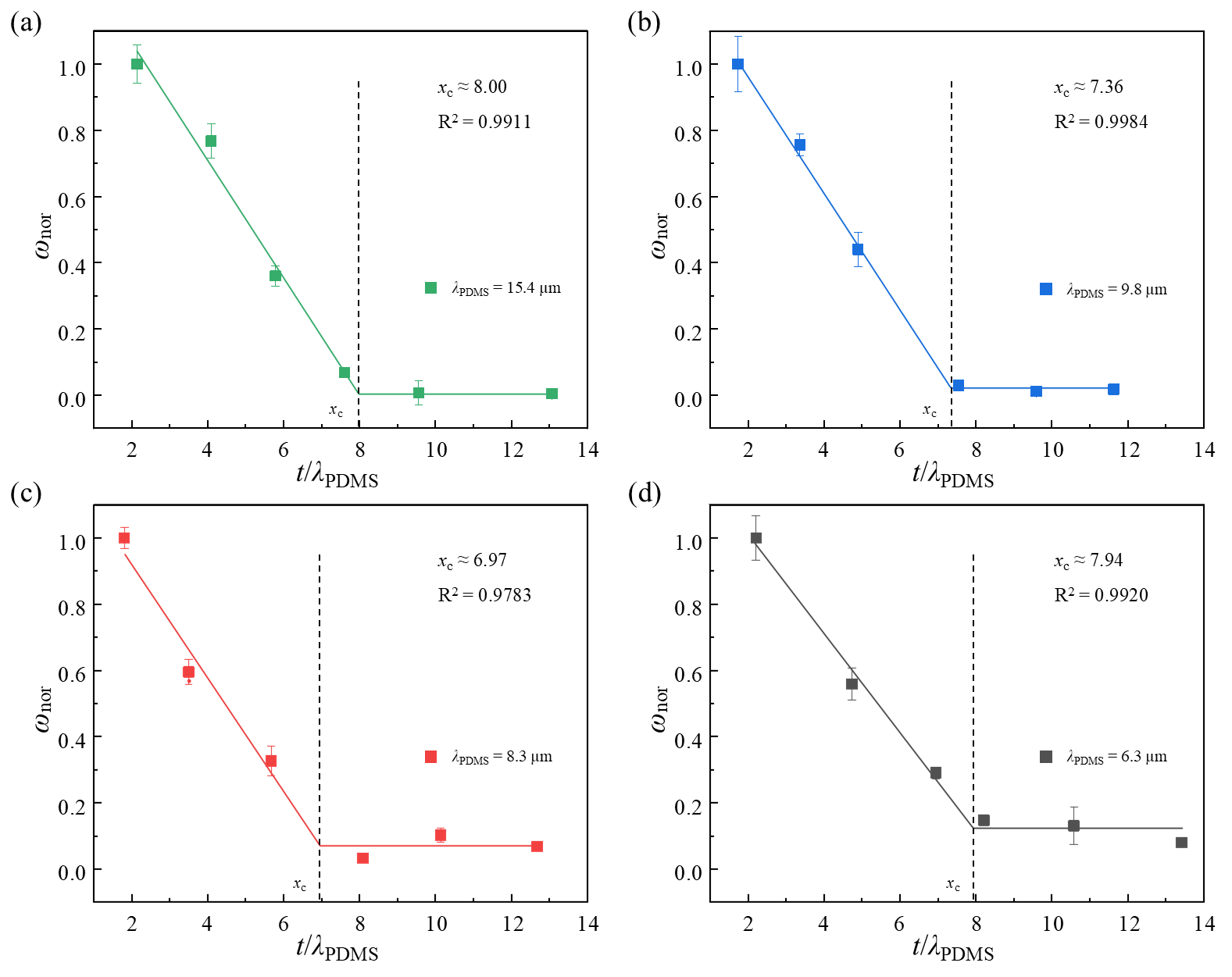
**

**Figure S4.** Linear-plateau regression results of the ASF experiment for normalized vorticity (*ω*_nor_) as a function of *t*/*λ*_PDMS_ at (a) *λ*_PDMS_ = 15.4 µm (*f* = 70 MHz), (b) *λ*_PDMS_ = 9.8 µm (*f* = 110 MHz), (c) *λ*_PDMS_ = 8.3 µm (*f* = 130 MHz), (d) *λ*_PDMS_ = 6.3 µm (*f* = 170 MHz).

**Linear plateau regression model:**

The model is a statistical model that describes a linear relationship where the response increases linearly and remains constant as the independent variable increases. After a certain point (breakpoint), the response stops linear phase increasing and reaches a plateau phase where the response remains constant. The quadratic plateau regression model can be written as stepwise functions defined below:

$$y=\left\{ \begin{matrix} a+bx for x\leq x_{c} \\ a+bx_{c} for x\geq x_{c} \end{matrix} \right.$$

where $y$ the response variable, $x$ is the predictor variable, *a* is the intercept, *b* is the slope during the linear phase, and the *x*_c_ is the breakpoint where the model reaches a plateau phase. For the linear plateau regression model to the ATH results, the coefficients of determination (R^2^) were calculated to be > 0.9, indicating the linear-plateau regression fitting showed good agreement with the experiment data.
